# Intraprostatic injection of botulinum toxin A: A promising treatment for patients with benign prostatic hyperplasia

**Published:** 2009

**Authors:** S. K. Agrawal, M. M. Agarwal, S. K. Singh

**Affiliations:** Department of Urology, PGIMER, Chandigarh, India E-mail: shrawanksingh2002@yahoo.com

## SUMMARY

In this open-labeled study, 77 men with BPH received an intraprostatic injection of 200 units of Botulinum toxin A (BT-A, Botox) using an ultrasound-guided transperineal approach. The American Urological Association (AUA) score, serum prostate-specific antigen (PSA), prostatic volume, residual volume, and peak urinary flow rates were evaluated before and after treatment at 1, 2, 6, 12, 18, 24, and 30 months follow-up. The primary endpoint was symptomatic improvement (AUA score) and peak urinary flow rates. The secondary endpoint was the evaluation of prostatic volume, serum PSA, and residual urinary volume.

At an evaluation after 1 month, 41 patients had subjective symptomatic relief. Compared with baseline values, the AUA score was reduced from 24.1 ± 4.6 to 12.6 ± 2.9 (*P* = 0.00001) and serum PSA was reduced from 6.2 ± 1.7 to 4.8 ± 1.0 ng/mL (*P* = 0.03). At the same time, the prostatic volume and residual urine volume were reduced by 12.7% and 12.8%, respectively and the mean peak urinary flow rate increased from 8.6 ± 2.9 to 13.1 ± 4.0 (*P* = 0.01). At an evaluation after 2 months, 55 patients had subjective symptomatic relief. The AUA score was reduced by 63.9% (*P* = 0.00001) compared with baseline values. In the same patients, serum PSA, prostatic volume, and residual urine volume were reduced by 51.6% (*P* = 0.00001), 42.8% (*P* = 0.00001), and 55.9% (*P* = 0.002), respectively and the mean peak urinary flow rate increased significantly. No local or systemic complications were observed after the treatment and none of the patients required narcotic analgesia after the procedure.

At the evaluation after 30 months, all 77 patients continued to have good voiding without worsening of LUTS. Their AUA symptoms score was 11.1 ± 2.7 (*P* = 0.02 *vs*. 2-month value) and total PSA was 3.1 ± 0.7 ng/mL (*P* = 0.7 *vs*. 2-month value).

Authors concluded that intraprostatic Botulinum toxin injection is a promising approach that is safe and effective.

## COMMENT

Recently, there have been many reports of use of BT-A in cases of symptomatic BPH.[1–3] It has been reported to induce apoptosis of prostatic stromal as well as epithelial cells and to decrease smooth muscle contractility.[3–4] Therefore, intraprostatic BT-A injection is likely to be effective in patients with BPH including those with a small prostate.[2]

Maria, *et al*.[1] pioneered the use of BT-A intraprostatic injections in patients with BPH. They reported a 50% decrease in prostate volume and significant symptomatic improvement in 13 out of 15 patients. Based on the present study, it appears that all patients with BPH are candidates for intraprostatic BT-A injection. The patient who doesn't receive any benefits with the first injection is likely to receive benefits with repeat injection. Significant improvements in subjective and objective parameters were observed in all 77 patients sooner or later. Although reinjection was required in 22, 14, and 7 patients at 2, 6, and 24 months of follow-up, improvement was seen in all the parameters after reinjection.

Minimally invasive options like TUMT, TUNA, etc. are usually offered to those with high risk for surgery; however these procedures have their own limitations. The BT-A therapy appears to be an attractive option even in high-risk patients with indwelling catheters or severe LUTS refractory to medical treatment. Recently Silva, *et al*.[5] reported that catheter removal was possible in 17 out of 21 patients of refractory urinary retention who were high-risk or had advanced malignancy.

Although it is too early to this about the use of BT-A in normal risk patients, it seems logical. Medical therapies used today for treatment of BPH are costly, have side effects, and require the patient's compliance, whereas treatment with BT-A doesn't require compliance and its effect may last for more than 2 years, as reported in this study. Moreover, this therapy seems to be free of complications and can be offered as an office procedure. Like the other studies, reduction in prostate size up to 50% was seen in this study. Long-term benefits may be due to reduction in prostate size after BT-A injection.

Results from this and other studies[1–3,5] show that intraprostatic BT-A injection has a place in the treatment of symptomatic patients with BPH and it seems to be safe, effective, and durable. However, more trials and studies compared with other modalities of treatments are required to arrive at a definite conclusion. The cost of BT-A treatment and the availability of ultrasound and expertise will be a limiting factor in our country.
